# Relationship between internal root resorption and dens in dente

**DOI:** 10.4317/jced.56944

**Published:** 2020-08-01

**Authors:** Ruth Pérez-Alfayate, Montse Mercadé, Jorge Vera

**Affiliations:** 1 DDS, PhD. Department of Dentistry, Universidad Europea de Madrid, Madrid, Spain; 2 DDS, PhD. Department of Dentistry, Universitat de Barcelona, Barcelona, Spain. Researcher, IDIBELL Institute, Barcelona, Spain; 3 DDS, PhD. University of Tlaxcala, Mexico; Private Practice, Puebla, Mexico

## Abstract

**Background:**

The aim is to report the treatment and follow-up of three lateral incisors with internal root resorption and dens in dente as a possible cause for their development, managed by root canal treatment and apical obturation with MTA or gutta-percha.

**Case description:**

This case report presents three clinical cases in which dens invaginatus type 2 is shown as a potential cause for the development of internal root resorption. Two cases were filled with a MTA apical plug technique and one with gutta-percha, and all were follow-up through time.

**Practical implications:**

The incidence of the association of internal root resorption with dens invaginatus may be underestimated and should be studied.

** Key words:**Dens in dente, dens invaginatus, internal root resorption, Mineral Trioxide Aggregate, palatal invagination.

## Introduction

Dens invaginatus is one of the most common developmental anomalies, with a prevalence of around 0.3% to 10% (1), resulting in invagination of the enamel organ into the dental papilla before calcification of the teeth (2).

Oehlers, in 1957 (3), classified this anomaly into three basic types, in which treatment options and prognosis were different: types 1 and 2 are characterized by incomplete invaginations, while types 3a and b are characterized by more complete invaginations. Regarding incomplete invaginations, type 1 has a prevalence of approximately 79%, being the most common (4). Invagination remains confined to the crown and does not extend beyond the level of the external cementoenamel junction (3). In type 2, which is less frequent, 15% of invaginations extend into the root, ending as a blind sac without communicating with the periodontium (3).

Regarding internal root resorption (IRR), which is the progressive destruction of intraradicular dentin of the canal walls as a result of clastic activities (5), many injurious events such as trauma, caries and periodontal infection, heat, calcium hydroxide procedures, vital root resection, anachoresis, cracked teeth, orthodontic treatment, and idiopathic dystrophic changes within nonpathological pulps have been described as causes for damage to the predentin and protective odontoblastic layer that could lead to IRR (6). This event is a challenge for the clinician, not only for differential diagnosis, but for treatment difficulties and prognosis.

## Case Report

-Case 1

**Figure 1:**
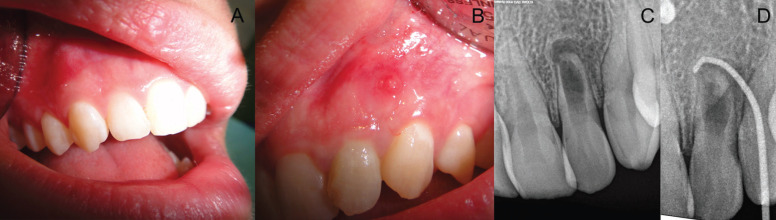
A. contralateral tooth showing a normal coronal anatomy; B. left lateral incisor showing a different incisal anatomy and the presence of a sinus tract; C. Periapical radiograph showing the presence of a dens invaginatus and an IRR; D. Radiograph with a gutta-percha cone trough the sinus tract pointing to the periapical lesion.

A 22-year-old, healthy female was referred for endodontic treatment of the maxillary left lateral incisor. The chief complaint was pain when biting and the presence of a sinus tract. The patient could not recall any causative traumatic event in her dental history. Changes in the occlusal anatomy were observed concerning the contralateral tooth (presence of palatal invagination and different incisal anatomy). A sinus tract between teeth #10 and #11 was observed, but the tooth was caries free (Fig. 1). Gingival probing depths were within normal limits. The tooth was sensitive to palpation and percussion and failed to respond to cold sensitivity testing, while the adjacent teeth all responded to the same tests within normal limits. Periapical radiographs demonstrated a radiolucent lesion in the apical third of the left lateral incisor consisting of a radiopaque image inside the crown resembling a dens in dente and a widening of the canal consistent with advanced internal root resorption (Fig. 1). Another radiograph was taken with a size 30 gutta-percha cone through the sinus tract, pointing to the apical region of this tooth. A clinical diagnosis was established of pulp necrosis with chronic apical abscess, dens invaginatus type 2, and internal root resorption. The primary purpose of treatment was to remove the infection and allow periapical healing.

**Figure 2:**
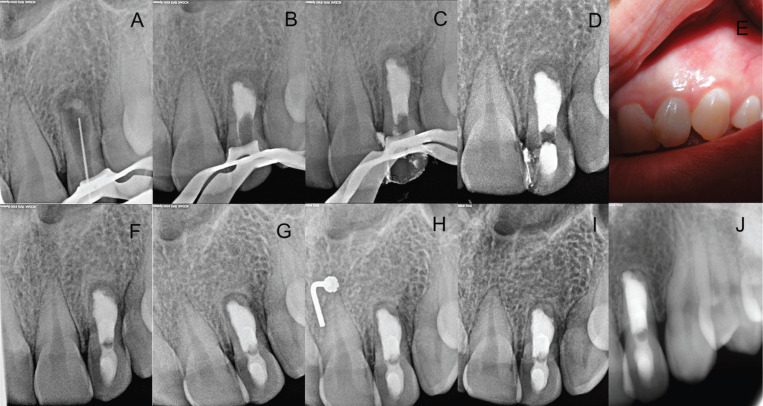
A. Working length X-ray determination; B. MTA apical plug; C. Gutta-percha backfill; D. Provisional restoration; E. Clinical follow-up at 6 months; F. Radiographic follow-up at 6 months; G. At 1 year; H. At 2 years; I. At 4 years; J. At 8 years.

After obtaining informed consent, the tooth was isolated with a rubber dam and access was gained to the pulp chamber, eliminating the invagination. Working length (WL) was established using a size 80 K-file and an electronic apex locator (Root ZX Mini, JJ Morita) to 17 mm (Fig. 2A). No instrumentation was applied so as not to further widen the canal, but irrigation with 5.25% sodium hypochlorite (NaOCl) solution was used. For the final irrigation, 1 mL of 17% EDTA (Irri-S; VDW) ultrasonically activated in three 20-second cycles, and a final irrigation with 5.25% NaOCl was performed. Root canals were dried with paper points size 80/.02 (Dentsply Maillefer). During the one-session treatment, mineral trioxide aggregate (MTA) was placed using ultrasonic activation of a plugger in the apical third of the canal, followed by backfill with gutta-percha (SuperEndo B&L-alfa, B&L Biotech) and a temporary filling of the access cavity (Cavit. 3M ESPE AG Dental Products) (Fig. 2B-D). The final restoration of the tooth was completed using composite in a second session, with follow-ups after 6 months, and 1, 2, 4, and 8 years (Fig. 2E-J).

-Case 2

**Figure 3:**
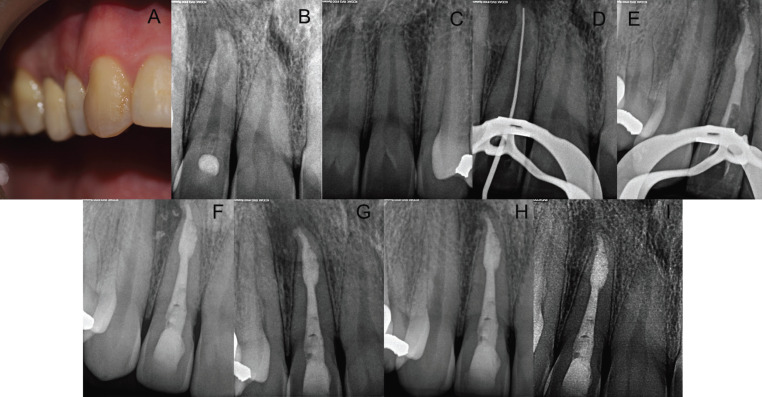
A. Clinical photography of the maxillary right lateral incisor. B. Initial radiograph of the Maxillary right lateral incisor; C. Maxillary left lateral incisor; D. Working length X-ray determination; E. MTA apical plug; F. Final restoration; G. Radiographic follow-up of tooth #7 at 5 months; H. At 2 years; I. At 3 years.

A 40-year-old, healthy male was referred for endodontic treatment of the maxillary right lateral incisor (Fig. 3A). Chief complaint was pain when biting. The patient could not recall any pertinent event in his dental history. Presence of a palatal restoration was observed during exploration, but the tooth was caries free. Gingival probing depths were within normal limits. The tooth was sensitive to palpation and percussion and failed to respond to cold sensitivity testing, while the adjacent teeth all responded within normal limits to the same tests. Periapical radiographs showed a radiolucent lesion in the apical third and a radiopaque image inside the crown resembling a dens invaginatus and a widening of the apical third of the canal. These were consistent with internal root resorption (Fig. 3B). The maxillary left lateral incisor showed normal anatomy (Fig. 3C).

A clinical diagnosis was established of pulp necrosis with apical periodontitis, dens invaginatus type 2, and internal root resorption. The primary purpose of treatment was to remove the infection and allow periapical healing.

After obtaining informed consent, the tooth was isolated with a rubber dam and access was gained to the pulp chamber, eliminating both the restoration and the invagination. WL was established using a size 15 K-file and an electronic apex locator (Root ZX Mini, JJ Morita) to 25 mm and instrumented with the Proper Next system (Dentsply Maillefer) to an apical size 30 (Figure 3D). A solution of 5.25% NaOCl was used during this session. Calcium hydroxide was placed and left for a second appointment.

After 15 days, during the second treatment session, irrigation with 5.25 % NaOCl was carried out; for the final irrigation, 1 mL of ultrasonically activated 17% EDTA (Irri-S; VDW) in three 20-second cycles, with a final irrigation with 5.25% NaOCl were used. Root canals were dried with paper points size 30/.02 (Dentsply Maillefer). MTA was placed using ultrasonic activation of a plugger in the apical third of the canal (17) followed by backfill with gutta-percha (SuperEndo B&L-alfa, B&L Biotech) and a final restoration of the access cavity with composite (Fig. 3E,F). The tooth was reexamined after 5 months, 2 years, and 3 years (Fig. 3G-I).

-Case 3

**Figure 4:**
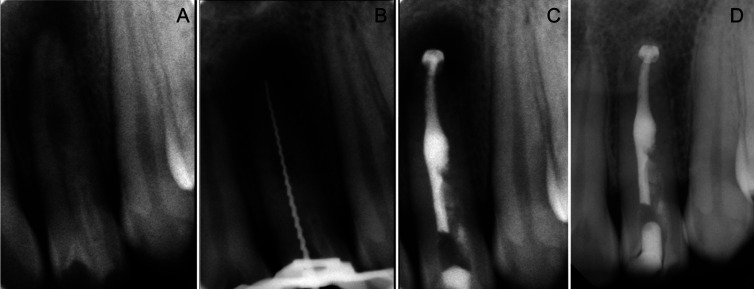
A. Initial X-ray. B. Working length X-ray determination; C. MTA apical plug and gutta-percha backfill; D. Radiographic follow-up at 8 years.

An 18-year-old, healthy female was referred for endodontic treatment of the maxillary left lateral incisor in 2011 (Fig. 4A). Chief complaint was pain when biting and on palpation at the buccal vestibule. A palatal groove was observed in the palatal surface but probing depths were all within normal limits and the tooth was caries free. The tooth was non-responsive to cold sensitivity testing while all other teeth that were tested responded within normal limits to the same tests. Periapical radiographs showed a radiolucent lesion in the apical and middle thirds of the root, a radiopaque image inside the crown resembling a dens-invaginatus and a widening in the middle third of the root canal consistent with internal resorption (Fig. 4A) The diagnosis was pulp necrosis with apical periodontitis, dens invaginatus type 2 and internal root resorption. After obtaining the informed consent, the tooth was isolated with a rubber dam and access was gained to the pulp chamber. WL was established using a size 20 H-file (Fig. 4B) and an electronic apex locator (Elements diagnostic, Sybron Endo, Orange CA) and instrumented with Twisted files (Sybron Endo) to an apical size 40/06. A solution of 5.25% NaOCl was used during this session. Calcium hydroxide was placed and left for a second appointment.

After 7 days, during the second treatment session, irrigation with 5.25 % NaOCl was carried out and activated with an ultrasonic tip at 2 mm from WL, then; for the final irrigation, 1 mL of 17% EDTA was used intracanal for 1 minute. Root canals were dried with sterile paper points and the canal was filled with continuous wave of condensation in the apical third, and then a back fill with the extruder and gutta-percha (Fig. 4C) using the Elements Obturation device (Sybron Endo). A final restoration of the access cavity with composite was done by the referral dentist. The patient returned 8 years later (Fig. 4D) for a consultation wishing to change the composite restoration on the tooth. She mentioned that she had remained asymptomatic ever since the root canal was finished. The patient was referred to have a new composite placed.

## Discussion

Many causes of damage to the predentin layer and the possible consequence of internal root resorption have been described in the literature, such as orthodontics, trauma, caries, and heat, among others (6). IRR is rare and insidious; its etiology and pathogenesis are only partially understood, but it is known that for IRR to occur, damage to the predentin and outermost protective odontoblastic layer must take place —usually occurring as a result of continuous, chronic inflammation — leading to exposure of the underlying mineralized dentin to odontoclasts (7). Nevertheless, information about dens in dente causing internal root resorption is scarce (8-10). Apparently, defects in the structure of the invagination or the enamel layer could lead to permeability or communication with the oral cavity (1), which produce damage to the predentin layer and chronic inflammation in the pulp, resulting in internal root resorption. Here, no findings that could lead to the development an IRR in the three clinical cases presented were found. A possible reason could be that pulpitis caused by the deep invagination of the three respective lateral incisors had caused this pathology. In a study by Kirzioglu *et al.* (8), the authors investigated the prevalence of dens invaginatus, observing this anomaly in 795 teeth of 2477 patients yet only one with internal root resorption. This fact suggests that resorption is extremely rare. Nevertheless, some of the causes of IRR described in the literature are classified as idiopathic. In a review conducted by Patel *et al.* (6), a clinical case of IRR is shown, but whether there was a concomitant dens in dente present was not mentioned, and the authors did not define other causes. A similar situation occurred in another publication by Tsurumachi *et al.* (11), in which the treatment of a dens invaginatus was explained. The presence of an apical/medium widening of the canal consistent with the existence of IRR was not described. On the other hand, it is nowadays possible to see some of these clinical cases on social media, though they are misdiagnosed. In this way, future studies on the association of these two pathological entities should be of interest. Evaluation of dens invaginatus types 1 and 2, even when asymptomatic, is recommended to prevent development of IRR and its consequences.

Ohelers, in 1957 (3), classified dens invaginatus as four types, based on clinical and radiographic findings. Although still used today, the classification system may lead to overlooking this anomaly. In the three clinical cases presented herein, radiographs were used to confirm the diagnosis. CBCT permits a better understanding of dens in dente because it helps determine whether the treated dens in dente is Type 1 or 2. In both cases, the prognosis and treatment options would be similar, so we decided that only two different radiographic projections were enough. Nevertheless, CBCT would help determine whether IRR communicated with the periodontal tissue, which would, of course, be relevant to the case prognosis (12).

The main objective of root canal treatment is to disinfect the root canal system to allow healing of the periradicular tissues. This should be followed by obturation with an appropriate root-filling material to prevent reinfection. In this sense, IRR defects can be difficult to obturate (6). Some studies (13,14) have demonstrated that warm gutta-percha is more effective in filling the defects than are cold techniques. In situations wherein the root canal wall has communicated with the periodontal tissue, MTA is recommended (6). Ultrasonic placement of this material has been shown to provide appropriate sealing. For these reasons, two of the lateral incisors were obturated at the IRR defect with MTA.

The association of IRR with dens invaginatus is an extremely rare finding and its incidence may be underestimated, adding to the difficulty of endodontic treatment. Thus, more research is needed to find a relation between these two pathologies.
